# Incidence of early neurodegenerative disease pathology in a forensic cohort

**DOI:** 10.1002/alz.091025

**Published:** 2025-01-03

**Authors:** Mikayla L Hunter, Hannah R Williamsen, Benjamin J Cooper, Samantha R Pierson, Catherine A Marcinkiewcz, Marco M Hefti, Kimberly L Fiock

**Affiliations:** ^1^ The University of Iowa, Iowa City, IA USA; ^2^ University of Iowa, Iowa City, IA USA

## Abstract

**Background:**

It is increasingly apparent that tau pathology in Alzheimer’s disease (AD) begins in the brainstems of middle‐aged patients, decades before the onset of symptoms. Most studies are, however, based on brain‐bank cohorts and focus on patients dying of natural causes. The true incidence of tau pathology in the brainstem thus remains unclear. In this study, we report a systematic characterization of Alzheimer’s disease and related dementia (ADRD) pathology in the brainstems of a statewide cohort of patients coming to forensic autopsy.

**Method:**

Postmortem human brain samples were obtained from asymptomatic forensic deaths in the state of Iowa (n = 12; 6 male and 6 female). Formalin‐fixed, paraffin embedded tissue samples were stained by immunohistochemistry (IHC) on the dorsal raphe (DR), locus coeruleus (LC), and hippocampus (HPC) to assess the presence of tau (AT8), α‐synuclein, and beta amyloid pathology. All stains were independently evaluated by two experienced neuropathologists, with discrepancies resolved by consensus.

**Result:**

We identified a total of 12 cases in our initial cohort (median age 51, range 25‐74). Six of the 12 cases contained tau pathology in the HPC, five of 12 contained tau in the DR, and five of 12 contained tau in the LC. Additionally, one case contained beta amyloid in neocortex and alpha synuclein in the DR. All cases positive for pathology were greater than 50 years of age.

**Conclusion:**

Our data suggests that incidental ADRD pathology is extremely rare in younger adults, with alpha‐synuclein and beta‐amyloid only seen in a single 74‐year‐old patient. These findings suggest such pathology is less common than previously thought and emphasizes the unique advantage of studying it in forensic cohorts dying of non‐natural causes, which better represents the population as a whole.

